# Association of Vitamin D Status with Lower Limb Muscle Strength in Professional Basketball Players: A Cross-Sectional Study

**DOI:** 10.3390/nu12092715

**Published:** 2020-09-05

**Authors:** Do Kyung Kim, Geon Park, Liang-Tseng Kuo, Won-Hah Park

**Affiliations:** 1 Department of Sports Medicine Center, Samsung Medical Center, Sungkyunkwan University School of Medicine, Seoul 03063, Korea; hrmax1@naver.com (D.K.K.); Geon2.park@samsung.com (G.P.); 2 Sports Medicine Center, Department of Orthopaedic Surgery, Chang Gung Memorial Hospital, Chiayi 613, Taiwan; 3 School of Medicine, College of Medicine, Chang Gung University, Taoyuan 333, Taiwan

**Keywords:** vitamin D insufficiency, muscle strength, basketball, athletes

## Abstract

Vitamin D deficiency in athletes may play a role in influencing fracture risk and athletic performance. This study aimed to examine the vitamin D status of basketball players and determine its correlation with muscle strength. We included 36 male professional basketball players (mean age, 22.6 ± 3.2 years) categorized by vitamin D status. We examined the muscle strength of knee extension/flexion and ankle dorsiflexion/plantarflexion using an isokinetic dynamometer. Eleven (30.5%), fifteen (41.7%), and ten (27.8%) players had deficient (<20 ng/mL), insufficient (20–32 ng/mL), and sufficient vitamin D levels (>32 ng/mL), respectively. In the dominant side, there were no significant correlations of vitamin D level with knee extension/flexion strength (*r* = 0.134, *p* = 0.436; *r* = −0.017, *p* = 0.922, respectively), or with plantarflexion/dorsiflexion ankle strength (*r* = −0.143, *p* = 0.404; *r* = 1.109, *p* = 0.527, respectively). Moreover, the isokinetic lower limb strengths were not significantly different between the three groups in all settings (all *p* > 0.05). In conclusion, professional basketball players had a high prevalence of vitamin D insufficiency. Though it may not be associated with muscle strength, maintaining adequate vitamin D levels by micronutrients monitoring, regular dietician consultation, and supplementation is still a critically considerable strategy to enhance young athletes’ health.

## 1. Introduction

Vitamin D is an essential hormone for calcium and phosphate metabolism and, hence, influences bone homeostasis and muscle function [1]. The synthesis of vitamin D relies on skin exposure to ultraviolet radiation B (UVB) in sunlight [2]. UVB exposure is moderate since the latitude of South Korea ranges from 33° N to 38° N; however, vitamin D deficiency is common in Korea [3]. Due to seasonal variation of UVB exposure, the vitamin D level of Koreans was lower in winter and spring [3]. According to previous studies, risk factors for vitamin D deficiency in Korea included living in urban areas, lacking exercise, working indoors, and being younger (20–49 years), especially for those who used sunscreen daily [3,4]. Prevention of skin aging and maintenance of youthful skin were critical factors associated with sunscreen use in young Koreans, regardless of sex [4].

Vitamin D deficiency can result in muscle loss and weakness [5]. Severe vitamin D deficiency can cause myopathy, accompanied by muscle weakness, amyotrophy, and muscle pain [6,7]. Vitamin D is essential for muscle function. Vitamin D may enhance muscle function by synthesizing protein in muscles and optimizing muscle growth while improving nerve-muscle function [8,9]. Optimal serum 25-hydroxyvitamin D (25(OH)D) helps to optimize overall performance for athletes and enhance muscle contraction [10]. For athletes, vitamin D is important not only for exercise performance but also for recovery [11]. Previous literature revealed that an insufficient vitamin D level was associated with an increased incidence of muscle damage [12], decreased athletic ability [13,14,15], and lower restorative ability after training and competition [11,16,17,18].

Vitamin D is essential to musculoskeletal health and exercise performance; however, vitamin D deficiency is not uncommon in the general population. According to a Korean national database study [3,19], the prevalence of vitamin D deficiency (<20 ng/mL) in 2014 was 75.2% in males and 82.5% in females. However, the cut-off value for vitamin D deficiency in Korea was higher than that of western countries [20,21]. Furthermore, the scene of vitamin D deficiency was also common among athletes. In a study on 279 NBA players, 79.3% of players had vitamin D insufficiency or deficiency, with 90 having vitamin D deficiency (<20 ng/mL) (32.3%) and 131 having insufficiency (20–32 ng/mL) (47.0%) [22]. According to a recent meta-analysis, 44–67% of the athletes were vitamin D inadequate (<32 ng/mL) [12].

Research on the effect of vitamin D on athletes has been gathering interest due to its potential role in improving athletic performance since enhanced muscle function is essential in boosting performance and reducing injuries for athletes [23,24]. However, studies on the effects of vitamin D for athletes are conflicting [14,25,26,27]. Some studies reported that vitamin D supplements in athletes with insufficient levels of vitamin D could increase quadricep strength and enhance vertical jump and sprint performance [14,25]. In contrast, other studies showed that vitamin D levels were not associated with muscle strength and motor ability [26,27]. Therefore, the association between vitamin D level and muscular performance in athletes remains uncertain. Our previous work showed that vitamin D insufficiency was common in Korean elite volleyball players, although their shoulder muscle strengths were not affected by low vitamin D status [28]. Little information about the vitamin D status and its association with lower limb muscle strength in professional basketball players is available.

Thus, we performed this study to investigate the vitamin D status of professional basketball players who participate in one of the most popular indoor sports and to evaluate the relationship between vitamin D concentration and extension/flexion strength of knees and plantar/dorsiflexion strength of ankles, both of which are critical components in jumping motions during a basketball game.

## 2. Materials and Methods

### 2.1. Participants and Demographics

We enrolled healthy male professional basketball players from the Samsung Thunders in the Korean Basketball League (KBL) by using the convenience sampling technique in this cross-sectional study from January 2015 to June 2017. The participants were routinely medically evaluated, healthy athletes and cleared for participation by an orthopedic specialist. All participants in this study regularly underwent training, including team-specific training supervised by coaching staff five times a week for at least four hours per day. During the two months of pre-season training and the regular season, the players were provided with a controlled diet under a nutritionist’s supervision. The controlled diet met the basic requirements for micronutrients including calcium and vitamin D. No other additional supplements including omega-3s and vitamin D were given. We excluded players who had undergone major knee and ankle surgeries and those already taking vitamin D supplements. All research procedures were reviewed and approved by the bioethical committee of the University of Sungkyunkwan. The study conformed to the tenets of the Declaration of Helsinki for medical research involving human subjects (IRB file No: 2020-04-199). All participants received a clear explanation of the study, including the risks and benefits of participation, and they provided written informed consent for testing and data analysis before the beginning of the study.

### 2.2. Assessments

All participants received an examination for serum vitamin D levels and muscle strength of their lower limbs in April (off-season period). The participants were instructed to fast overnight for at least 8 h and were abstained from any vigorous physical activity and exercise at least 24 h before the assessments to avoid the confounding effects of post-exertional muscle fatigue. After arriving in the laboratory, the fasting serum samples (3 mL) were taken from the antecubital vein area of the arm and collected into a tube. The collected blood samples were clotted for 30 min at room temperature (20–22 °C), and centrifuged at 3000 rpm (revolutions per minute) for 15 min. The separated serum samples were stored at −70 °C until analysis. All archived samples of the discovery cohort were sent to the Department of Laboratory Medicine at the Samsung Medical Center, and vitamin D levels were determined from a single baseline serum sample. Serum levels of 25(OH)D2 and D3 were determined by high-performance liquid chromatography-tandem mass spectrometry detection (Euroimmun AG, Lübeck, Germany). All assays met reproducibility requirements of ≤20% coefficient of variation (CV) and were acceptable for clinical use. Total vitamin D levels were quantitated using calibration curves constructed from the mass chromatogram.

There is no universally accepted standard definition for vitamin D deficiency, insufficiency, or sufficiency. In our study, vitamin D levels were defined as deficient at <20 ng/mL, insufficient at 20–32 ng/mL, and sufficient at >32 ng/mL, which were described by Fishman et al. [22]. We used an isokinetic dynamometer (CSMI Medical Solutions, MA, USA) to evaluate the knee and ankle muscle strengths of all participants. This testing protocol was conducted on the dominant and non-dominant legs of each subject. The dominant leg was determined by which hand is dominant. Musculoskeletal physiotherapists performed standardized testing under the supervision of one of the authors. After warm-up using a stationary bike for 10 min, the participants performed three submaximal familiarization trials. Thereafter, they underwent maximal concentric tests. The participants were given verbal support to encourage maximal effort. Concentric knee extension/flexion peak torques were measured at angular velocities of 60°/s. Next, the participants performed ankle dorsiflexion/plantarflexion muscle strength tests at a speed of 30°/s. The maximum peak torque (Nm) for each velocity was also recorded.

### 2.3. Statistics

Statistical analysis was performed to evaluate the correlation between player parameters and vitamin D levels using Pearson correlation coefficients. We also calculated the concentric extension/flexion and dorsiflexion/plantarflexion muscle strengths of the dominant side and analyzed the isokinetic strength with respect to vitamin D level using one-way analysis of variance (ANOVA). The level of statistical significance was set at 0.05. One-way ANOVA and a post-hoc Bonferroni test was used to analyze the data. Statistical analysis was conducted using SPSS ver 18.0 (SPSS Inc., Chicago, IL, USA)

## 3. Results

We included 36 participants in this study. The mean age of the athletes was 22.6 ± 3.0 years. The mean vitamin D level was 24.7 ± 7.2 ng/mL. Regarding vitamin D levels, there were 11 (30.5%), 15 (41.7%), and 10 (27.8%) players who were deficient, insufficient, and sufficient, respectively. Twenty-six players (72.2%) were either vitamin D deficient or insufficient (Table 1).

Correlation between vitamin D and knee and ankle strengths revealed no significant findings. There was no significant bivariate correlation between vitamin D and extension/flexion knee strength of the dominant side at 60 deg/sec (*r* = 0.134, *p* = 0.436; *r* =−0.017, *p* = 0.922, respectively). Similarly, we found no significant correlation between vitamin D level and isokinetic ankle plantarflexion/dorsiflexion strength of the dominant side at 30 deg/sec (*r* = −0.143, *p* = 0.404 and *r* = 0.109, *p* = 0.527, respectively) (Table 2). In the non-dominant side, no significant correlations were noted between vitamin D level and knee or ankle isokinetic strengths (all *p* > 0.05, Table 2).

**Figure 1:**
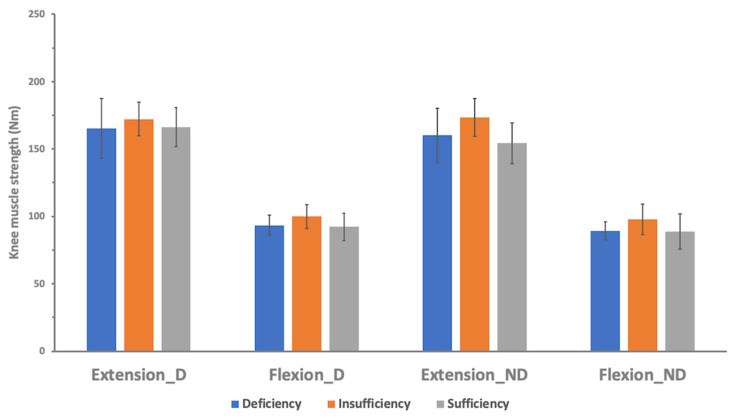
Isokinetic knee muscle strength at 60°/s according to vitamin D status. The isokinetic knee strengths were not significantly different across the three groups in all settings (Extension_D, knee extension at dominant side; Flexion_D, knee flexion at dominant side; Extension_ND, knee extension at non-dominant side; Flexion_ND, knee flexion at non-dominant side).

**Figure 2:**
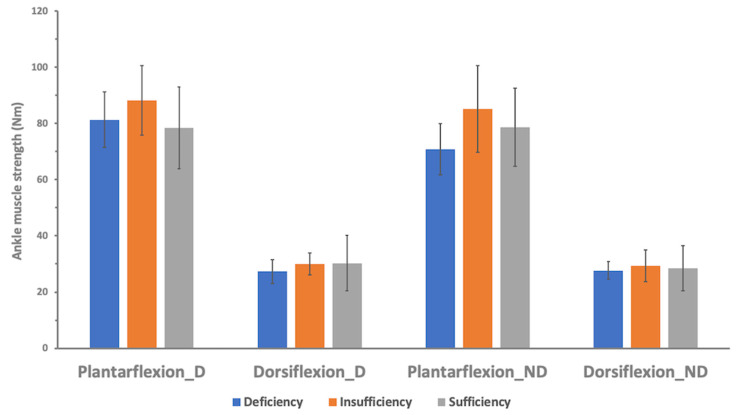
Isokinetic ankle muscle strength at 30°/s according to vitamin D status. The isokinetic ankle strengths were not significantly different across the three groups in all settings (Plantarflexion_D, ankle plantarflexion at dominant side; Dorsiflexion_D, ankle dorsiflexion at dominant side; Plantarflexion_ND, ankle plantarflexion at non-dominant side; Dorsiflexion_D, ankle dorsiflexion at non-dominant side).

The participants were divided into three groups by the level of vitamin D to examine maximal muscle strength differences according to vitamin D levels. There were no significant differences in knee and ankle maximal concentric isokinetic muscle strengths between the three groups, neither dominant nor non-dominant sides (Table 3 and Table 4, Figure 1 and Figure 2).

## 4. Discussion

The present study examined the serum concentration of vitamin D and its effect on Korean professional basketball players’ muscle strength. The most important finding of this study was that most of the basketball players were either vitamin D insufficient or deficient, although the vitamin D status did not significantly affect the lower limb muscle strength.

In a study on NBA players, 79.3% had vitamin D insufficiency (20~32 ng/mL) (47.0%) or deficiency (<20ng/mL) (32.3%) [22]. Similar to our results, the findings of this study showed that the majority of professional basketball players lack vitamin D. According to a meta-analysis, which surveyed 2,313 professional athletes playing various sports, 44–67% of the athletes were vitamin D inadequate (<32 ng/mL) [12]. The vitamin D synthesis relied on skin exposure to UVB radiation in sunlight [2]. Therefore, the choice of an indoor or outdoor training environment influences sun exposure, ultimately affecting vitamin D synthesis. Emerging evidence has indicated that athletes who train outdoors have higher vitamin D levels than those who train indoors or avoid peak daylight hours, regardless of latitude or season [29]. In this study, the professional basketball team trained five times a week (for more than five hours), but the athletes spent most of their training time at an indoor gym. That may be why most of the participants in this study had vitamin D deficiency or insufficiency. Previous literature also shows that basketball players have a relatively lower vitamin D level than other outdoor athletes [26,30].

There may not be a universal cut-off value for optimal vitamin D status. A blood 25(OH)D concentration below 10 ng/mL or 12 ng/mL is considered the lower limit of vitamin D status and an indicator of risk of vitamin D deficiency [20,21]. The World Health Organization (WHO) has also defined vitamin D “insufficiency” as a serum 25(OH)D below 20 ng/mL and “deficiency” as a serum 25(OH)D below 10 ng/mL [20]. In this study, we took a recommendation from the American Nutrition Society as a reference. It recommended that vitamin D deficiency be defined as a 25(OH)D level of 20 ng/mL or less [31], which was echoed in the consensus for optimal serum 25(OH)D concentrations from Central Europe, including Poland, Hungary, Belarus, Estonia, Czech Republic, and Ukraine [32]. This cut-off level was also utilized in various previous studies [3,7,19,28,33], including the Korean national survey [3,19] and our previous study [28].

Vitamin D is essential for athletes because it reduces injury rates, is useful in skeletal muscle repair and remodeling [34], and aids in efficient muscle recovery before vigorous-intensity exercises [18,34,35]. Moreover, vitamin D affects muscle tissue, primarily by increasing the size and quantity of type II (fast-twitch) muscle fibers [34,36,37]. Close et al. [14] reported that increasing vitamin D intake for eight weeks in athletes decreased 10 m sprint times and enhanced exercise abilities, such as vertical jumps [14,38]. Wyon et al. also found a significant increase in some of the muscle strength measurements [39]. However, in a study on vitamin D levels and lower limb muscle strength in isokinetic exercise among professional soccer players, there was no association between lower limb muscle strength and vitamin D levels [27].

Furthermore, Todd et al. found that the prevalence of vitamin D deficiency can be resolved by oral supplementation, but muscle function and respiratory function did not improve after that [40,41]. Brännström et al. found no significant correlation between these parameters, including jump and sprint performance, and vitamin D levels [42]. Other studies also reported that the associations of muscle strength and physical performance with vitamin D in athletes could not be adequately explained [36,43]. The results of research examining the association between serum vitamin D concentration and muscle strength and function in athletes have been contradictory. Moreover, not accounting for physical activity level or body composition change [14,39] or lacking a suitable priori power calculation [27,42] made interpretation of the above findings difficult. Since the current study was a cross-sectional design, we could not imply a dose-response of vitamin D on muscle function. The difference of results in the present study from the other cross-sectional Polish study indicating a positive relationship between vitamin D and muscle function [43] might come from the difference of baseline vitamin D levels from different exposure amounts of UVB in different latitudes. Meanwhile, the training environments in different kinds of sports might also play a role.

We measured the muscle strength of knee extension/flexion and ankle plantarflexion/dorsiflexion using isokinetic equipment to evaluate the muscles typically utilized while jumping, a significant movement in basketball. While the application of constant angular velocities does not necessarily characterize performance in sports, the evaluation of muscle strength using isokinetic equipment is valid, reliable, and widely used in the assessment of muscle function in athletics [44].

The findings of the current cross-sectional study did not find a relationship between vitamin D deficiency and impaired lower limb strength in professional basketball players. There were two explanations for these findings. The first is the appropriateness of the isokinetic test. Some studies have shown that vitamin D deficiency results in a reduction in type II muscle fibers [45]. Type II fibers produce faster muscle contractions and provide greater strength than do type I fibers; therefore, the performance of explosive and nimble movements such as sprinting, jumping, and turning is closely related to the contraction of type II muscle fibers [46].

However, accurate functional assessment of fast-twitch fibers (type II), used for vertical jumps, may not have been achieved since the evaluation of lower extremity strength by using isokinetic equipment in the current study was more consistent with the evaluation of type I fibers than that of type II fibers. As a result, there is insufficient data to assess young and healthy basketball players’ athletic abilities with only isokinetic test results.

The second one is the physical well-preparedness of the participants. The participants included in this study were elite athletes who were already highly skilled and may have little room for muscle strength improvement. The effect of long-term training may potentially overcome the negative effect of vitamin D deficiency on muscle. Meanwhile, the current assessment tools may be unable to detect the tiny difference, and thus, sensitive and standardized measurement techniques for athletes are urgently needed.

There were still some limitations to this study. First, the external validity of the findings was limited. The results of the current investigation would be difficult to apply in other settings, since only male Korean basketball players were included within the analysis. Second, this is a cross-sectional study where the vitamin D levels were only examined one time. Third, other potential confounding factors for calcium and vitamin D levels, including diet, degree of sun exposure, individual training hours/modality, and sunlight practice, were not completely controlled. Although we only included young, healthy athletes and excluded those with diseases or medications that might affect vitamin D, bias should be considered while applying the findings of the current investigation. Fourth, a convenience sample was used in this study. Although we enrolled almost all players on a single professional basketball team, the condition of the participants may not be representative of players in other groups. A future study involving multiple teams may be needed to validate the present findings.

## 5. Conclusions

The current study showed that more than two-thirds of young professional basketball players had vitamin D insufficiency or deficiency. Although this is not associated with lower limb muscle weakness, maintaining an adequate vitamin D level by micronutrients monitoring, regular dietician consultation, and supplementation is still a critically considerable strategy to enhance the young athletes’ health and performance. Future study investigating the effect of vitamin D on athletic performance should be performed under more critical control of the potential confounders, such as sunlight exposure and dietary intake.

## Figures and Tables

**Table 1:** Players’ demographics by vitamin D status.

Variable	Vitamin D Level			*p* Value ^a^
	Deficiency (<20 ng/mL)	Insufficiency (20–32 ng/mL)	Sufficiency (>32 ng/mL)	
No. players (%)	11 (30.5)	15 (41.7)	10 (27.8)	
Vitamin D (ng/mL)	16.4 ± 3.2	24.6 ± 2.6	33.9 ± 1.4	<0.001 *
Age (yr)	22.3 ± 3.2	22.3 ± 3.3	23.2 ± 2.4	0.731
Height (cm)	187.5 ± 7.2	188.5 ± 5.5	192.8 ± 8.9	0.198
Weight (kg)	82.8 ± 7.8	83.5 ± 7.1	85.6 ± 8.8	0.697
Body mass index (kg/m^2^)	23.3 ± 1.4	23.4 ± 1.5	22.1 ± 1.5	0.098

Values are presented as mean ± standard deviation. ^a^ *p* value for between group comparisons, and the significance level was set as 0.05. * *p* < 0.05.

**Table 2:** Correlation coefficients (r) between vitamin D level and other characteristics.

Characteristics	Vitamin D (ng/mL)	*p* Value
Age (yr)	0.045	0.796
Height (cm)	0.227	0.184
Weight (kg)	0.077	0.656
BMI (kg/m^2^)	−0.295	0.080
Dominant side		
Knee		
Extension	0.134	0.436
Flexion	−0.017	0.922
Ankle		
Plantarflexion	−0.143	0.404
Dorsiflexion	0.109	0.527
Non-Dominant side		
Knee		
Extension	−0.058	0.737
Flexion	−0.056	0.748
Ankle		
Plantarflexion	−0.014	0.934
Dorsiflexion	0.028	0.871

Abbreviation: BMI, body mass index.

**Table 3:** Comparison of maximal isokinetic knee strength by vitamin D level.

Knee Strength at 60°/s (Nm)	Vitamin D Level			*p* Value ^a^
	Deficiency (<20 ng/mL)	Insufficiency (20–32 ng/mL)	Sufficiency (>32 ng/mL)	
Dominant				
Extension	165.3 ± 33.0	172.3 ± 22.7	166.2 ± 20.5	0.753
Flexion	93.5 ± 11.012214	99.9 ± 16.0	92.2 ± 14.1	0.340
Non-Dominant				
Extension	160.1 ± 30.3	173.3 ± 25.2	154.2 ± 21.5	0.182
Flexion	89.0 ± 10.1	98.0 ± 20.5	88.8 ± 18.6	0.316

Values are presented as mean ± standard deviation. ^a^ *p*-value for between-group comparisons, and significance level was set as 0.05.

**Table 4:** Comparison of maximal isokinetic ankle strength by vitamin D level.

Ankle Strength at 30°/s (Nm)	Vitamin D level			*p* Value
	Deficiency (<20 ng/mL)	Insufficiency (20–32 ng/mL)	Sufficiency (>32 ng/mL)	
Dominant				
Plantarflexion	81.4 ± 14.7	88.3 ± 22.4	78.5 ± 20.3	0.445
Dorsiflexion	27.3 ± 6.2	30.0 ± 7.1	30.3 ± 13.8	0.694
Non-Dominant				
Plantarflexion	70.8 ± 13.6	85.2 ± 27.8	78.7 ± 19.4	0.277
Dorsiflexion	27.7 ± 4.6	29.3 ± 10.2	28.4 ± 11.2	0.901

Values are presented as mean ± standard deviation.
